# Managing the Complication of Band Erosion in Banded Sleeve Gastrectomy: A Case Report

**DOI:** 10.1007/s11695-023-07041-z

**Published:** 2024-01-11

**Authors:** Mohamed Hany, Ahmed Zidan, Anwar Ashraf Abouelnasr, Mohamed Ibrahim, Bart Torensma

**Affiliations:** 1 Department of Surgery, Medical Research Institute, Alexandria University, 165 Horreya Avenue, Hadara, Alexandria, 21561 Egypt; 2 Madina Women’s Hospital, Alexandria University, Alexandria, Egypt; 3 Clinical Epidemiologist, Leiden University Medical Center (LUMC), Leiden, The Netherlands

**Keywords:** Revision surgery, Sleeve gastrectomy, Erosion, Banded procedure, Gastrogastrostomy

## Abstract

We present a case involving a patient with laparoscopic banded sleeve gastrectomy (BSG) with a 3-month history of persistent vomiting, decreased tolerance for fluids, and limited intake of soft food items. Upon investigation, an eroded band and gastric dilatation were identified. The treatment involved the removal of the eroded band and a segment of the stomach, followed by the restoration of gastric continuity through a gastrogastrostomy.

**Supplementary Information:**

The online version contains supplementary material available at 10.1007/s11695-023-07041-z.

## Introduction

The introduction of Banded Sleeve Gastrectomy (BSG) aimed to address the issue of long-term weight regain observed following Sleeve Gastrectomy (SG). A systematic review (SR) indicated superior weight loss outcomes with BSG, demonstrating a margin of + 6.39% and + 9.97% in percentage total weight loss (%TWL) at 3 and 5 years postoperatively, respectively, compared to SG [1]. The incidence of band erosions associated with the placement of a ring in BSG seems to be a not frequently published complication, as it has not been reported in studies specifically addressing BSG [2, 3]. Across three systematic reviews comparing BSG and SG, there were no documented cases of band erosions or perforations [1, 4, 5]. In contrast, a systematic review focusing on Banded RYGB reported incidences of band erosion and band slippage at rates of 2.3% and 1.5%, respectively [6].

The correct placement of a band occurs after peri-gastric dissection, positioned 4–5 cm from the gastroesophageal junction. A band of size for males is 7.5 (diameter of 24 mm) and for females is 7.0 (diameter of 22 mm) and should be placed loosely around the pouch. Management of band erosion can be addressed either through endoscopic intervention or a surgical approach.

## Case Presentation

A 23-year-old female presented at the Medical Research Institute, Alexandria University, Egypt, with a 3-month history of persistent vomiting. She complained of decreased tolerance and intake of fluids and soft food items. Nutrients described before surgery were a soft diet with a fortified protein equivalent to 20 g and an oral nutrition solution of 15 g twice daily. Currently, her weight was 45 kg, resulting in a BMI of 16.5 kg/m^2^, (in the last 3 months, she lost almost 12 kg). Laboratory values were within the normal range, except for a low hemoglobin level of 10.1 g/dl and an albumin level of 3.4 g/dl.

In August 2022, she underwent BSG, with an unknown type of band/ring placement, at another clinic. Preoperatively, she weighed 105 kg, corresponding to a BMI of 38.56 kg/m^2^.

Computed Tomography (CT) with 3D reconstruction imaging was employed, confirming slippage and complete band erosion, and the sleeve pouch above the band had become dilated. Furthermore, slippage and erosion of the band were hindering endoscopic passage.

Endoscopic extraction of the band was unsuccessful. Following this, two surgical approaches were proposed by the patient and family—either the removal of the eroded band along with a segment of the stomach, followed by RYGB reconstruction, or the restoration of gastric continuity through gastrogastrostomy.

## Operation

The original surgical ports were reused, and adhesiolysis commenced at the site where the fully eroded band was not visible. The segment of the sleeve containing the eroded band was excised using the Echelon 60-mm black reloads (Ethicon, Cincinnati, OH, USA). Subsequently, continuity was restored; a gastrogastrostomy was created by inserting one arm of the stapler into the distal portion and the other into the proximal portion laterally, utilizing the Echelon 60-mm gold reload. The stoma was then closed with a V-lock 180 PDS 3/0 barbed suture (Medtronic, Mansfield, MA) to complete the gastrogastrostomy. Intraoperative endoscopy was conducted to confirm patency and assess for any leaks. A drain was strategically placed along the pouch. Postoperatively, the patient demonstrated early tolerance to oral fluids, beginning on the first day after surgery without any complications. The patient was subsequently discharged on the second postoperative day and the drain was removed. At the 2-month follow-up, the patient reported no instances of vomiting, returned to the normal diet, and gained 3 kg.

## Conclusion

Gastrogastrostomy may be considered a suitable option when the band has eroded inside the stomach and endoscopic retrieval has failed. This approach is particularly beneficial for frail patients who may not be able to withstand more intensive surgical procedures, such as revisional RYGB. The selection of an appropriate surgical strategy should be individualized based on patient-specific factors.

### Supplementary Information

Below is the link to the electronic supplementary material.

Supplementary file1 (MP4 244863 KB)

## Data Availability

Data can be requested from the corresponding author.
